# Structural and Nanotribological Properties of a BODIPY Self-Assembly

**DOI:** 10.3389/fchem.2021.704915

**Published:** 2021-08-06

**Authors:** Shanchao Tan, Wendi Luo, Yongjie Zhang, Xiang-Kui Ren, Yuhong Liu, Zhijian Chen, Qingdao Zeng

**Affiliations:** ^1^ State Key Laboratory of Tribology, Tsinghua University, Beijing, China; ^2^ CAS Key Laboratory of Standardization and Measurement for Nanotechnology, CAS Center for Excellence in Nanoscience, National Center for Nanoscience and Technology (NCNST), Beijing, China; ^3^ Laboratory of Theoretical and Computational Nanoscience, CAS Key Laboratory of Nanophotonic Materials and Devices, CAS Center for Excellence in Nanoscience, Beijing Key Laboratory of Ambient Particles Health Effects and Prevention Techniques, National Center for Nanoscience and Technology, Chinese Academy of Sciences, Beijing, China; ^4^ School of Chemical Engineering and Technology, Tianjin University, Tianjin, China; ^5^ Center of Materials Science and Optoelectonics Engineering, University of Chinese Academy of Sciences, Beijing, China

**Keywords:** self-assembly, bodipy dye, scanning tunneling microscopy, nanotribology, hydrogen bond

## Abstract

Boron-dipyrromethenes (BODIPY) are promising functional dyes, whose exceptional optical properties are closely related to their supramolecular assembly. Herein, the self-assembly of a BODIPY derivative functionalized with uracil groups is explicitly and thoroughly investigated by using scanning tunneling microscopy (STM). Based on the simulation and calculation by density functional theory (DFT) method, it can be concluded that the construction of ordered self-assembly structure is attributed to the formation of hydrogen bonds between uracil groups. Moreover, the nanotribological property of the self-assembly on HOPG surface is measured by using atomic force microscopy (AFM). The effort on self-assembly of the BODIPY derivative could enhance the understanding of surface assembly mechanism.

## Introduction

Since the first discovery in 1968 by Treibs and Kreuzer (Treibs and Kreuzer, 1968), boron dipyrromethene (BODIPY) has received increasing research interest as a class of excellent fluorophores (Ulrich et al., 2008; Boens et al., 2012). The BODIPY core can be extended further by grafting different functional groups without significantly changing its fluorescent properties, which makes the BODIPY derivatives easy to acquire and apply (Loudet and Burgess, 2007; Gupta et al., 2019). In addition to the advantages of high extinction coefficients and high quantum yields, BODIPY is reasonably stable to physiological environment because of the insensitivity to the polarity and pH conditions (Karolin et al., 1994; Loudet and Burgess, 2007). Therefore, BODIPY dyes have been widely applied in a variety of fields including fluorescence labeling agents (Madhu et al., 2013; Kim et al., 2016; Baxter and Wittenberg, 2019), chemosensors (Atilgan et al., 2010; Wang et al., 2015; Yu et al., 2016), fluorescent switches (Golovkova et al., 2005; Moreno et al., 2016), photovoltaics/optoelectronics (Lee and Hupp, 2010; Bura et al., 2012; El-Khouly et al., 2014) and so on.

Due to the rigid aromatic backbone, BODIPY dyes have an inherent ability to form assemblies and aggregates, which has been studied and discussed a lot in recent years (Cherumukkil et al., 2018). The self-assembly of an amphiphilic BODIPY derivative synthesized by Yang et al. (Yang et al., 2015) shows comparable optical characteristics with classical cyanine dyes, which could be attributed to the highly sliding 2D herringbone-type packing. Rödle et al. (Rodle et al., 2017) demonstrated hierarchical assembly of temperature-controlled H-type aggregating BODIPY dyes, driven by *π-π* stacking interaction, amide-based hydrogen bonding and strong hydrophobic effect. Matarranz et al. (Matarranz et al., 2018) have reported the self-assembly of the BODIPY dye functionalized with a butyric acid group. The supramolecular assembly structures in the solid state are formed by the stacking of translational BODIPY units through *π-π* stacking and hydrogen bonding interactions. However, most researches on self-assembly of BODIPY can only indirectly acquire the structure of aggregates by spectroscopic methods.

**FIGURE 1:**
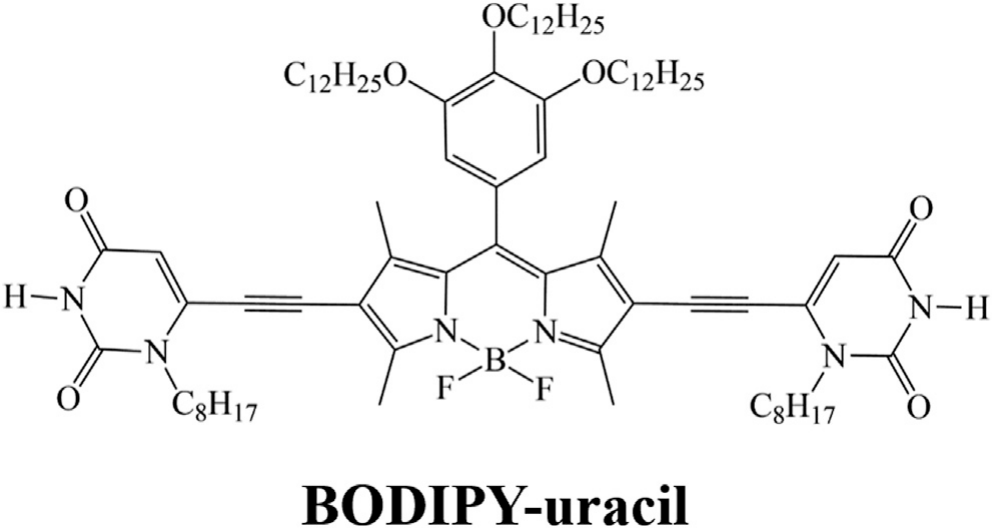
Chemical structure of BODIPY-uracil.

In this work, the self-assembly of a BODIPY dye, namely BODIPY-uracil, was deeply investigated on highly oriented pyrolytic graphite (HOPG) surface. BODIPY-uracil is a BODIPY derivative synthesized by Zhang et al. (Zhang et al., 2020), as shown in Figure 1. This BODIPY dye could polymerize into J-aggregates through intermolecular hydrogen bonds and feature resonant fluorescence, showing potential application in organic light emitting devices. Herein, the explicit self-assembly structure of BODIPY-uracil on HOPG surface was directly characterized by using STM to further investigate the assembly mechanism. Moreover, the DFT calculation and AFM measurement were conducted to deeply explore the structural and nanotribological properties.

## Results and Discussion

### Structural Characterization

**FIGURE 2:**
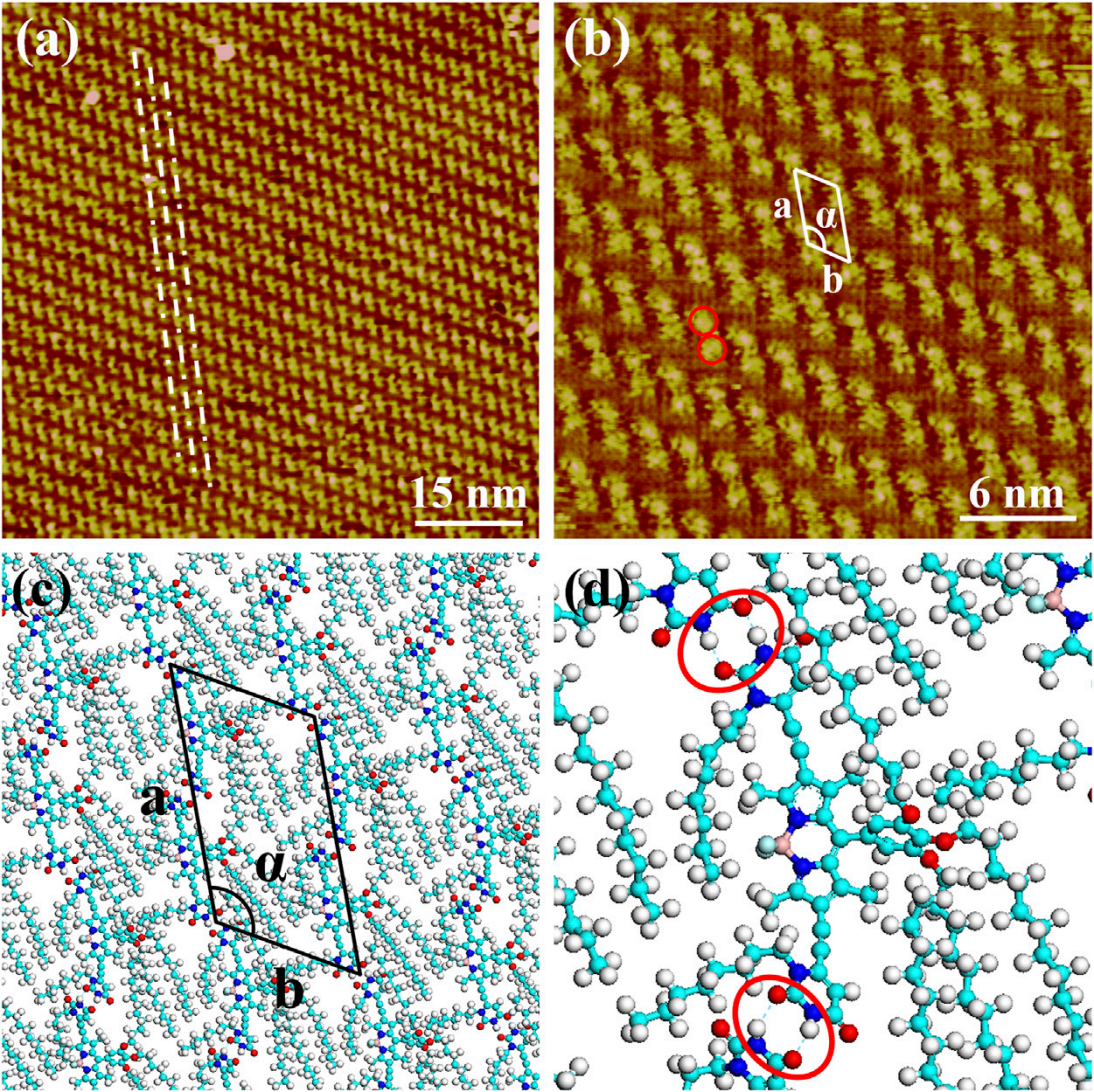
STM images of the self-assembly structure of BODIPY-uracil at the heptanoic acid/HOPG interface: **(A)** large-scale **(B)** high-resolution. Tunneling conditions: **(A)** *I*_set_ = 110.20 pA, *V*_bias_ = 960.60 mV; **(B)** *I*_set_ = 85.27 pA, *V*_bias_ = 960.60 mV. **(C)** The simulated molecular packing structures. **(D)** The enlarged simulated molecular packing structure.

The nanoarchitecture of BODIPY-uracil at the heptanoic acid/HOPG interface is characterized by STM, verifying the formation of hydrogen bonds between uracil groups. As shown in the large scale STM image in Figure 2A, bright rods are aligned into the parallel rows, as marked with white dash-and-dot lines. Further structural details are presented in Figure 2B, revealing that each rod element consists of two bright spots (as marked with red circles), whose measured average diameter is 0.8 ± 0.1 nm, showing good agreement with the theoretical size of the BODIPY core. Therefore, it can be concluded that the rod element is corresponding to the dimer composed of two end-to-end BODIPY-uracil molecules, while the uracil groups and alkyl chains cannot be clearly recognized owing to the lower density of electric states. The BODIPY-uracil molecule can interact with molecules above and below in the same row through N-H···O hydrogen bonding between uracil groups, as marked with red circles in the enlarged simulated molecular packing structure in Figure 2D. Besides, the stretched alkyl chains, as shown in the simulated molecular packing structure in Figure 2C, can also interact with each other through the van der Waals force. Therefore, the assembly motif of BODIPY-uracil derives from both the hydrogen bonding interaction between uracil groups and van der Waals interaction between alkyl chains. Besides, the BODIPY-uracil molecules are adsorbed on the HOPG surface through the *π-π* stacking interaction between BODIPY dyes and substrate, which also plays an important role in the formation of stable self-assembly. The parameters of the unit cells overlaid in Figure 2B are measured as follows: *a* = 3.9 ± 0.1 nm, *b* = 2.3 ± 0.1 nm, α = 119 ± 2°.

### Theoretical Calculation

The theoretical size and interaction energy of BODIPY-uracil self-assembly are calculated using DFT method based on the STM characterization. The calculated unit cell parameters are listed in Table 1, which are in accordance with the STM measurement. The interaction energies in the self-assembly are also calculated, as listed in Table 2. Herein, the lower interaction energy indicates the stronger interaction. It is revealed that the intermolecular interaction energy (−72.848 kcal/mol) is much higher than the interaction energy between assembled molecules and the substrate (−130.317 kcal/mol). Therefore, it can be concluded that the *π-π* stacking interaction between BODIPY-uracil molecules and HOPG substrate is stronger than the hydrogen bonding interaction between BODIPY-uracil molecules.

**TABLE 1:** Experimental (Expt.) and calculated (Cal.) cell parameters of BODIPY-uracil on the HOPG surface.

		a (nm)	b (nm)	α(°)
BODIPY-uracil	Expt	3.9 ± 0.1	2.3 ± 0.1	119 ± 2°
	Cal	4.1	2.4	120

**TABLE 2:** Total energies and energies per unit area of self-assemblies on the HOPG surface.^a^

	Interaction between molecules (kcal mol^−1^)	Interaction between molecules and substrate (kcal mol^−1^)	Total energy (kcal mol^−1^)	Total energy per unit area (kcal mol^−1^ Å^−2^)
BODIPY-uracil	−72.848	−130.317	−203.164	-0.244

a The total energy includes the interaction energies between molecules and the interaction energies between molecules and substrate.

### Nanotribological Measurement

**FIGURE 3:**
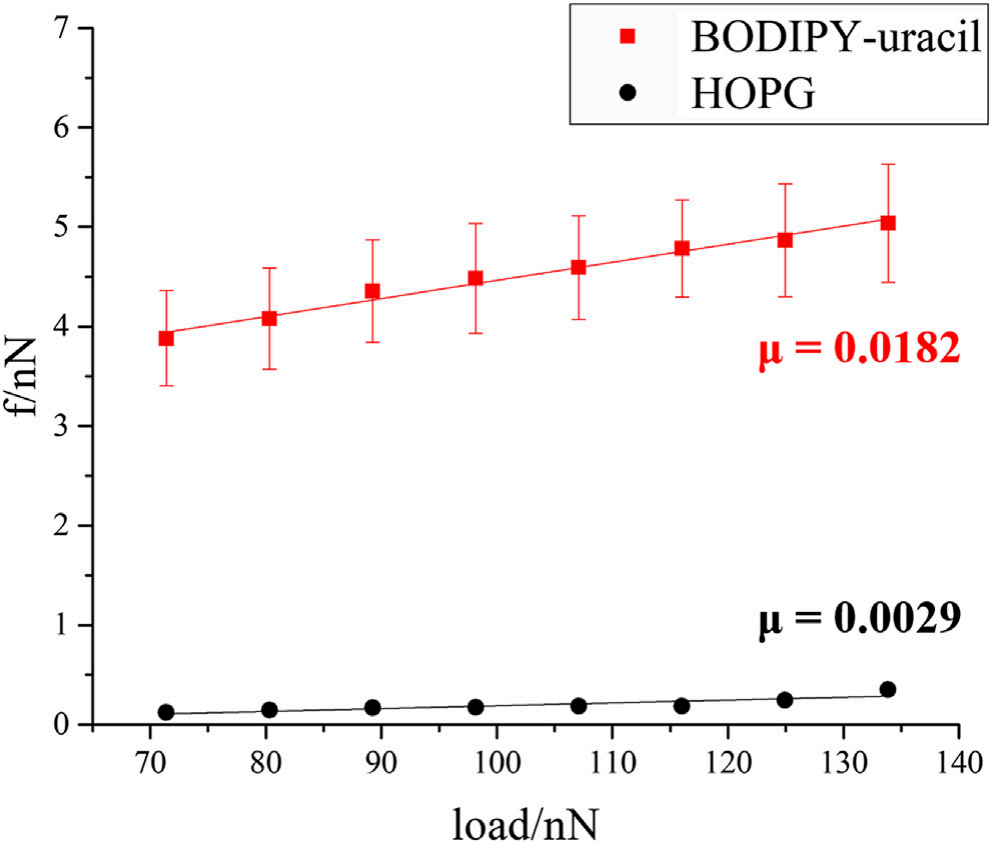
Friction force as a function of applied load of BODIPY-uracil and HOPG.

To investigate the nanotribological property of BODIPY-uracil, the friction forces are measured by using AFM. Figure 3 shows the friction forces of BODIPY-uracil and HOPG as a function of applied load. The friction-load data are linearly fitted so that the friction coefficient *μ* can be acquired as the slope of the linear fitting curve, as labeled in Figure 3. Obviously, the friction forces and friction coefficient have increased significantly after the construction of BODIPY-uracil self-assembly on HOPG surface, which is due to the stronger interaction between silicon tip and functional groups of BODIPY-uracil than that between tip and inorganic substrate. Besides, the surface topography and electron cloud density of BODIPY-uracil assembly is much rougher, so the energy barrier the tip needs to overcome is much higher during the frictional process, resulting in the high friction coefficient of BODIPY-uracil self-assembly.

### Conclusion

To sum up, the structural and nanotribological properties of a BODIPY self-assembly were thoroughly investigated at nanoscale. The linear assembly structure of BODIPY-uracil at solid/liquid interface was clearly and directly observed by using STM. The DFT calculation and simulation demonstrated the formation of hydrogen bonds between uracil groups, which is the primary assembly motif of the ordered nanoarchitecture. Besides, the nanotribological property of the self-assembly on HOPG surface is characterized by using AFM. This work of BODIPY self-assembly enhances the understanding of assembly mechanism.

## Materials and Methods

The BODIPY-uracil was synthesized according to the reported methods (Zhang et al., 2020). The structure of self-assembly was characterized by using a Nanoscope IIIA STM system (Bruker, Germany) under ambient conditions. The microscopic friction forces were measured using an MFP-3D AFM (Asylum Research, United States). And the theoretical calculations were performed using DFT-D scheme provided by DMol3 code. Further experimental and theoretical details can be seen in the Supplementary Material.

## Data Availability

The raw data supporting the conclusion of this article will be made available by the authors, without undue reservation.
