# Supplementary material for: Structural and Nanotribological Properties of a BODIPY Self-Assembly
Source: Front Chem. 2021 Aug 6;9:704915. doi: 10.3389/fchem.2021.704915 (PMC8377353; doi:10.3389/fchem.2021.704915)
Supplement: Supplementary file 1 [file DataSheet1.docx]

Supplementary Material

# Materials and Sample Preparation

The BODIPY-uracil was synthesized according to the reported methods (Zhang et al., 2020). Heptanoic acid used as the solvent was purchased from J&K Chemical Ltd. (Beijing, China). All the chemicals above were used without any further purification. The BODIPY-uracil is dissolved in heptanoic acid with the concentration less than 10^-4^ M. Then the self-assembly sample was prepared by depositing a droplet of the solutions (0.1 μL) onto the freshly cleaved HOPG (grade ZYA, NTMDT, Russia) surface. All the solution and assembly sample were prepared under ambient conditions.

# STM characterization

The samples were characterized using a Nanoscope IIIA system (Bruker, Germany) under ambient conditions. To achieve better imaging resolution of the assemblies, the measurements were carried out at the liquid-solid interface by immersing the STM tip directly into the solution. All the images were captured with the mechanically made from Pt/Ir (80:20) wires under constant-current mode. The figure captions have included the specific tunneling conditions (i.e., tunneling current and bias). The drift for all the images was calibrated using an atomic-resolution HOPG lattice as a reference. The experimental cell parameters were obtained by using the length measurement function of the STM software “*NanoScope (R) III Digital Instrument*” (Veeco Instrument Inc., America). After selecting two or three STM images, five groups of cell parameters were measured in each image and all the data were averaged to figure out the final cell parameters.

# DFT calculation

Theoretical calculations were performed using DFT-D scheme provided by DMol3 code. We used the periodic boundary conditions (PBC) to describe the 2D periodic structure on the graphite in this work. The Perdew-Burke-Ernzerhof parameterization of the local exchange correlation energy was applied in the generalized gradient approximation (GGA) to describe exchange and correlation (Perdew et al., 1996). All-electron spin-unrestricted Kohn-Sham wave functions were expanded in a local atomic orbital basis. For the large system, the numerical basis set was applied. All calculations were all-electron ones, and performed with the medium mesh. Self-consistent field procedure was done with a convergence criterion of 10^-5^ au on the energy and electron density. Combined with the experimental data, we have optimized the geometry of the adsorbates in the unit cell. When the energy and density convergence criterion were reached, we could obtain the optimized parameters and the interaction energy between adsorbates. To evaluate the interaction between the adsorbates and HOPG, we design the model system. Since adsorption of benzene on graphite and graphene should be very similar (MacLeod et al., 2015), we had performed our calculations on infinite graphene monolayers using PBC. Considering that the interaction between adsorbates and substrate was mainly van der Waals interaction, the Grimme’s dispersion corrections were adopted in our calculations. In the superlattice, graphene layers were separated by 35 Å in the normal direction. When modeling the adsorbates on graphene, we used graphene supercells and sampled the Brillouin zone by a 1x1x1 k-point mesh.

# Friction force measurements

The microscopic friction forces of BODIPY-uracil were measured using an MFP-3D AFM (Asylum Research, America) at room temperature. To eliminate the solvent effect on the friction forces, the measurements were conducted at the gas-solid interface after the solvent was evaporated. The silicon CSG10 probe with a rectangular cantilever (nominal normal spring constant 0.1 N m^-1^) was used. All the friction pairs are silicon probe (CSG10) – sample (assembly on HOPG). It should be mentioned that the genuine normal and lateral factors need to be calibrated before each measurement. The normal photodetector sensitivity was calculated from the slope of the force curve obtained on a hard substrate. The normal spring constant was estimated from the power spectral density of thermal noise fluctuations under ambient conditions (Hutter and Bechhoefer, 1993). By scanning a commercially available TGF11 silicon grating (MikroMasch, Estonia), the lateral factor was calculated based on an improved wedge calibration method so that the voltage results can be transformed into the force values (Ogletree et al., 1996; Varenberg et al., 2003). The microscopic friction forces were measured by scanning in the perpendicular direction to the cantilever in the contact mode. The scan size was 160 nm × 160 nm, and the scan rate was 1 Hz. During the measurements, the feedback gains should be appropriate to make sure that the noise of the deflection signal was minimal while the height signal was tracked well. At least three positions were measured for each sample. For the data processing, friction values were calculated as half of the difference between the trace and retrace signals, such that the topography effect on friction can be excluded.

# Reference

Hutter, J.L., and Bechhoefer, J. (1993). Calibration of Atomic-Force Microscope Tips. *Review of Scientific Instruments* 64(7)**,** 1868-1873.

MacLeod, J.M., Lipton-Duffin, J.A., Cui, D., De Feyter, S., and Rosei, F. (2015). Substrate Effects in the Supramolecular Assembly of 1,3,5-Benzene Tricarboxylic Acid on Graphite and Graphene. *Langmuir* 31(25)**,** 7016-7024.

Ogletree, D.F., Carpick, R.W., and Salmeron, M. (1996). Calibration of frictional forces in atomic force microscopy. *Review of Scientific Instruments* 67(9)**,** 3298-3306.

Perdew, J.P., Burke, K., and Ernzerhof, M. (1996). Generalized gradient approximation made simple. *Physical Review Letters* 77(18)**,** 3865-3868.

Varenberg, M., Etsion, I., and Halperin, G. (2003). An improved wedge calibration method for lateral force in atomic force microscopy. *Review of Scientific Instruments* 74(7)**,** 3362-3367.

Zhang, Y.J., Liu, P., Pan, H.F., Dai, H.T., Ren, X.K., and Chen, Z.J. (2020). Alignment of supramolecular J-aggregates based on uracil-functionalized BODIPY dye for polarized photoluminescence. Chemical Communications 56(80), 12069-12072.
